# Progress of Surgery

**Published:** 1900-12-29

**Authors:** 


### PROGRESS OF SURGERY.

**Chronic Suppuration of the Middle Ear.**—A. S. Cobbledick[1] writes on this subject, his remarks applying to those cases of middle-ear disease which persist, with intermissions, for many years in spite of careful treatment. He points out that by reason of possible cranial complications it is necessary to cure these cases. Much can often be done for them without the radical operation. If meatal swelling and eczema exist this should be first carefully treated. Daily, for a week or ten days, the parts should be cleansed with warm boracic lotion, and the meatus then packed with a strip of cyanide gauze soaked in a lead and tar, or a lead and alum, lotion. The eczema improves under this treatment, and the meatal swelling diminishes. The possibility of the presence of a foreign body should always be borne in mind. It is shown that the presence of particles of cholesteatoma in the discharge points to de-definite treatment. In the treatment of polypi the method recommended is to first prepare the cases for three days by frequently irrigating with boracic lotion, and afterwards instilling rectified spirits. To diminish the troublesome bleeding during the operation for removal of the polypi, the use of a mixture of equal parts of 20 per cent. cocaine solution and 50 per cent. solution of supra-renal capsule is advocated. This is to be dropped and left in the ear ten minutes before operation. Then as much of the fluid as possible is to be mopped out to prevent a dangerous quantity of cocaine being swallowed after passing along the eustachian tube. The great bulk of the polypus can be removed with Wilde's aural snare, and the remains with a small ring or spoon curette. The curetting may be followed by complete facial paralysis if performed forcibly. If the polypi are recurrent they probably have origin around as equestrum in the attic region. Lalle's attic curette is a useful instrument for the removal of granulations and ossicular remains in that region. It is advised to soak the gauze plug used afterwards in iodoform emulsion, as it will then be much more easily removable than if applied dry. If, on examining the drums in these chronic cases, the tympanic portion of the membrane is almost intact, but a large perforation of Shrapnell's membrane exists, the condition indicates caries of the head of the malleolus or cholesteatoma, or both, to be the exciting cause of the discharge. Allusion is made to cases where severe attacks of pain are frequent, and are due to sclerosis of the mastoid process, and can only be relieved by opening the antrum. Cobbledick then points out the indications for the removal of the ossicles. This should be done for (1) the cases which resist less radical treatment; (2) those in which adhesion of the malleus and membrane to the tympanic wall interfere with drainage of the upper spaces; (3) cholesteatoma of the upper spaces; (4) cases of attic suppuration with much impairment of hearing; and (5) when, though suppuration has stopped, there is evidence of increased labyrinthine pressure. The technique of the operation is then described.

**Intranasal Treatment in Diseases of the Ear.**—Peter McBride[2] introduced a discussion on this subject at the annual meeting of the British Medical Association in Ipswich. He alluded to the marked benefit effected in some cases by the removal of post-nasal adenoids. Recurrent attacks of acute otitis media might be prevented by this treatment, and certain cases of chronic suppuration might be materially benefited. Of the former class, if the cases had not gone far, and if there were no complications such as hereditary syphilis, the effect of removing adenoids was often little less than marvellous. The drum membranes were usually indrawn, and often thickened, and there was a history of repeated colds, each leaving the patient more deaf than before. The operation might be very beneficial, even though the use of Politzer's bag failed to give any relief. The case of adults complaining of increasing hardness of hearing was then considered. The drum membranes in these cases might be normal, or indrawn, sometimes with patches of thickening or dark atrophic arras, or might show a red shade. Occasionally irregularity in the outline of the malleus was the only abnormality. The normal or nearly normal membrane was generally associated with a normal or nearly normal naso-pharynx, whereas the cases with indrawing, thickening, and atrophy of the drum-head were often associated with diffuse congestion of the upper respiratory tract. One form of progressive deafness (the sclerotic) was commonly due to changes in the region of the stapus, often associated with a lesion of the osseous capsule of the labyrinth, while the other was ascribed to chronic middle-ear catarrh. McBride's attitude with regard to intra-nasal operations was that no such operations should be undertaken except in the catarrhal cases when obvious nasal symptoms were present; and that in those cases where anchylosis of the stapes existed, with or without implication of the labyrinth, operations of any kind were liable to do harm. The catarrhal and the sclerotic types of ear disease were often mixed, and experience would have to be relied upon in such cases then to decide the question of operation. It was pointed out that nasal polypus often existed for years without ear trouble supervening, and the same was true of hypertrophic rhinitis, and nasal obstruction from septal causes. In adenoid cases it appeared that direct mechanical interference with the eustachian orifices might be the cause of the deafness, and if the adenoids were situated high up in the naso-pharynx away from these tubes deafness as a rule was not produced. Adenoids might affect hearing power by interference with the blood circulation in the middle ear, as well as with the ventilation of that cavity. David McKeown (2) points out that when adenoids are removed for deafness in a large number of cases the hearing is found to be much improved immediately after the operation. This improvement is usually followed by some temporary loss, due to the irritation following the operation. He then records a case which showed this immediate improvement, although the drum was perforated, in which, of course, it was clear that no alteration in the intra-tympanic air tension was produced. This points to the immediate improvement in all cases being the result of alterations in the circulation of the blood in the tympanum and labyrinth. Again, the final amount of improvement may not be the best obtained, perhaps because adhesions formed later between the posterior wall of the pharynx and the eustachian tubes.

**The Radical Mastoid Operation.**—Charles A. Ballance[3] has described a very elaborate and complete form of this operation for the cure of chronic purulent otorrhœa. He performs two operations, the first for the complete exposure and removal of the disease, and the second to promote rapid healing of the large bone wound which has been made. In acute profuse otorrhœa without mastoid signs, and in all cases of acute mastoid inflammation the antrum, or the antrum and any other suppurating mastoid cells, should alone be opened, the tympanum and meatus being left undisturbed. The meatus should not be interfered with in acute cases unless the mastoid has become infected from the posterior wall of the osseous meatus. The author points out the great importance, in operating for the cure of chronic cases, of removing the bridge of bone which overhangs the communication between the antrum behind and the attico-tympanal cartilage in front. The outer wall of the attic is formed by the bone of the roof of the osseous meatus, and it contains cells which communicate with those of the mastoid process and with the attic. If this operation is complete a bent probe placed in contact with the tegmen tympani can be withdrawn without meeting with any resistance. The posterior bony meatal wall has to be removed also, so that the tympano-antral-attic cavities will be easily accessible through the enlarged meatus. The healing of the bone from the bottom is usually secured by tamponing the cavity with strips of dry gauze. This is a painful proceeding, and has to be carried out for many months. To curtail this, Ballance has devised his second operation, which consists in reopening the wound and applying an epithelial graft or grafts to the bony cavity. *The first operation.*—The skin incision completely exposes the mastoid process. It starts half an inch in front of the meatus in the line of the hair. It follows the line of the hair until that line passes into the neck. It is then continued downwards and forwards to the posterior part of the apex of the mastoid. The incision is carried to the bone one-third of an inch from the margin of the flap. To remove the bone the instrument strongly recommended is a special electrical burr. Failing that gouges must be used, never a drill or trephine. The edge of the gouge should be at right angles to the shaft. The antrum can always be found by passing a bent probe backward into it from the attic. In curetting, the facial nerve is most liable to be damaged at the posterior part of the inner wall of the tympanum. A special method of dealing with the cartilaginous meatus is described. With a long narrow knife the inferior wall of the canal is divided vertically well into the concha, and the cut in the concha is curved upwards and backwards to the level of the beginning of the anterior helix. The posterior wall is then pushed upwards and backwards, and attached in a special manner with silk-worm gut to the mastoid flap, raw surface to raw surface, the thick layer of tissue behind this wall of the meatus having been cut away previously to diminish resiliency. After plugging the cavity in the bone the mastoid flap is sutured in place. *The second, the epithelial grafting operation,* may be done in children at the end of a week, in adults in ten days to a fortnight or three weeks. The flap is turned forward again, the cavity papered with the grafts, gold leaf being used as a protective, and again plugged. The plug is removed in a week. These operative methods of Ballance have the same results upon the hearing power as the older method of tamponing. The Eustachian tube may become sealed up, but the tube is not required either for ventilation or drainage after the operation. Notes of twenty cases subjected to this treatment are appended. In all the results were excellent.

**Fibroma Involving the Tympanum.**—E. W. Fleming (Los Angeles, Cal.)[4] reports a case of this kind. The patient, a man of forty, had noticed a feeling of fulness in the left ear for a month, and every day the sensation had become more intense. A few days after this symptom there was a slight discharge of pus, and a solid fleshy mass could be felt near the orifice of the meatus. Twelve days later the growth filled the auditory canal. When Fleming saw him there was a fairly solid, tumour-like mass growing outward and almost filling the concha. The visible portion was coarsely lobulated, and dark red in colour, with blood-vessels visible on the surface. It was tightly wedged in the canal, so that the point of origin could not be made out. A large piece of the growth was removed for microscopical examination with a cold-wire snare. It proved to be a true fibroma. A radical operation was refused. For six weeks the treatment consisted in the removal of fragments with the snare, and the application of the galvano-cautery. After two weeks' rest the tumour was again as large as at first. The patient was taken into hospital and access to the growth obtained by making an incision over the mastoid and displacing the pinna and cartilaginous canal forwards and incising the latter. It was now seen that the growth filled the tympanic cavity, and was attached to it as well as to the posterior wall of the bony canal. There was no evidence of the presence of a drum membrane. The mass was removed, and its apparent base scraped and cauterised. Two or three weeks later granulations became exuberant and filled the canal. Curettment was done and the parts rendered free and open again. When seen seven months after being first examined, the auditory canal was closed except for a small sinus through which muco-pus issued. There was no recurrence of the growth.

**Sarcoma of Mastoid.**—Stephen A. Lutz[5] relates the following case. A boy of nine and a half years of age had a growth in the auditory canal entirely obscuring the membrane, and had pain and offensive discharges. Some of the tissue was removed, and the base cauterised. In three days the canal was again filled. The mass was repeatedly removed, but recurred. Then the mastoid was opened, and found to contain a foul-smelling mass of granulations and pus. The cavity was carefully cleansed. Later the parotid gland became involved, and the growth from the mastoid continually increased in size. The left tonsil was pushed beyond the median line of the throat. The microscopical examination revealed a small, round-celled sarcoma, with a number of giant cells.

[1] Clin. Jour., Aug. 1900. [2] Brit. Med. Jour., Sept. 1900. [3] Medico-Chir. Trans., 1900 (Aug.). [4] Laryngoscope, July, 1900. [5] Laryngoscope, Aug. 1900 (abstract).

